# Editorial: Achieving nutrition security in Tanzania by improving production, education and economics: Methods, tools and applications

**DOI:** 10.3389/fpubh.2022.1014741

**Published:** 2022-09-20

**Authors:** Harry Konrad Hoffmann, Joyce Ludovick Kinabo, Stefan Sieber, Wolfgang Stuetz, Michelle Bonatti, Hadijah Ally Mbwana, Götz Bernhard Uckert, Custodio Efraim Matavel, Johannes Michael Hafner, Katharina Löhr, Constance Rybak

**Affiliations:** ^1^Sustainable Land Use in Developing Countries (SusLAND), Leibniz Centre for Agricultural Landscape Research (ZALF), Müncheberg, Germany; ^2^Department of Human Nutrition and Consumer Sciences, Sokoine University of Agriculture, Morogoro, Tanzania; ^3^Department of Agricultural Economics, Faculty of Life Sciences Thaer-Institute, Humboldt-Universität zu Berlin, Berlin, Germany; ^4^Institute of Nutritional Sciences, University of Hohenheim, Stuttgart, Germany; ^5^CIFOR-ICRAF, ICRAF Country Programme, Dar es Salaam, Tanzania; ^6^Urban Plant Ecophysiology, Humboldt-Universität zu Berlin, Berlin, Germany

**Keywords:** food security, nutrition security, Tanzania, East Africa, small-scale farmers, climate change, wood energy, gender

Globally, the number of people unable to afford a healthy diet rose by 112 million to almost 3.1 billion, reflecting the impacts of rising consumer food prices during the pandemic (1). In this context, food security and nutrition is increasingly highlighted as a priority among both scientists (2) and political decision makers (3).

On the African continent, this topic is of particular importance as the number of individuals affected by undernourishment remained on a high level throughout the 2010s, and since 2019 are again rising (Figure 1)– the war in Ukraine will most likely cause even greater problems (4). In 2021, hunger affected 278 million people in Africa.

**Figure 1:**
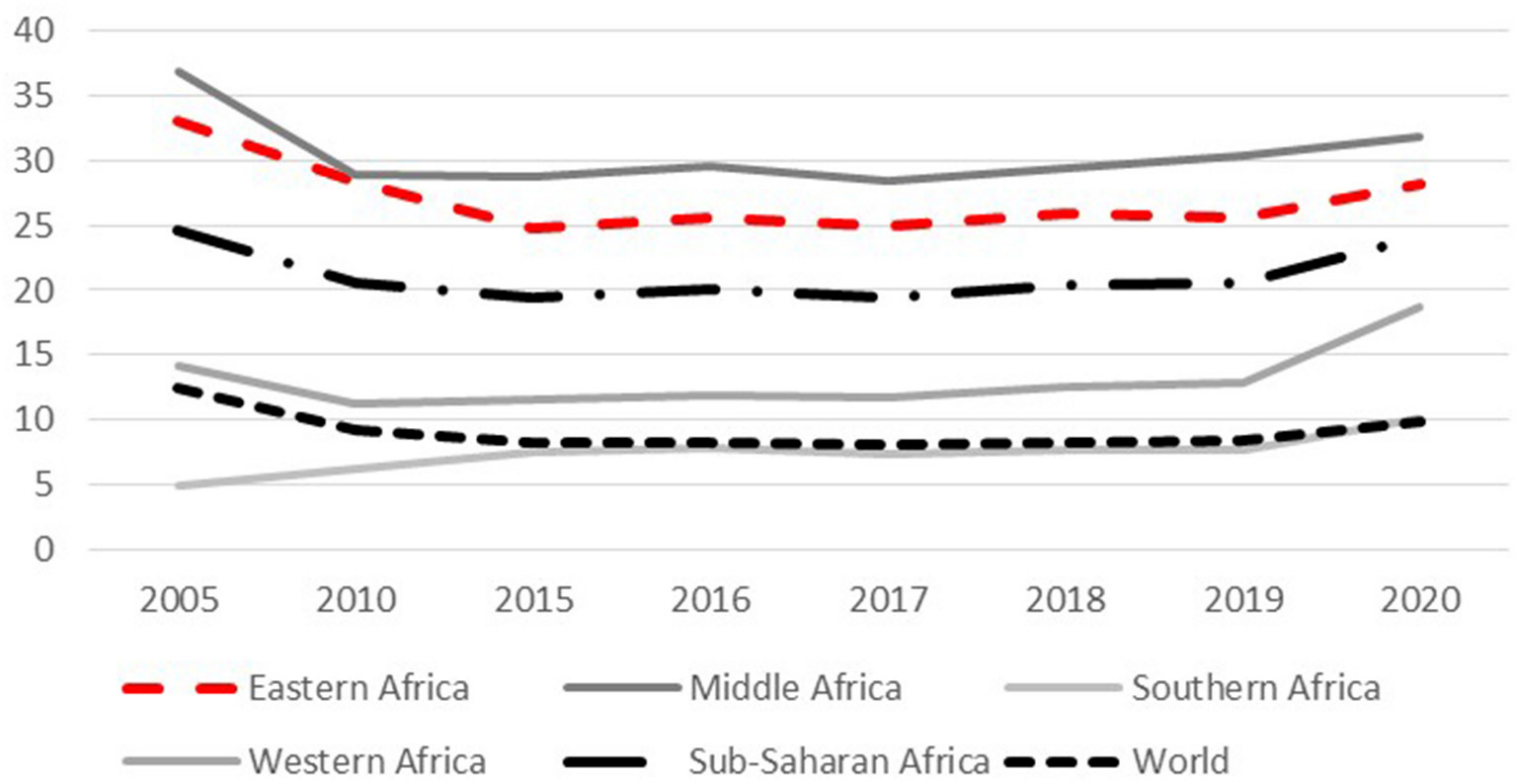
Prevalence of undernourishment in % of population (5).

Situated in one of the globally most affected regions, achieving food security and nutrition is already a substantial challenge in Tanzania, with the Food and Agriculture Organization (FAO) (5) reporting that, between 2014 and 2020, the overall prevalence of moderate or severe food insecurity in the total population has affected roughly 55-56% of the overall population. However, these already severe numbers do not display the food security situation in 2022 adequately because the devastating effects of the Covid-19 pandemic (6) and the subsequent spikes in global food prices since the 24th of February 2022 are not included. The latter is especially worrying as more than 65% of imported wheat in Tanzania derives from Russia (ca. 50%) or Ukraine (ca. 15%) (7). Furthermore, prices for agricultural inputs, like fertilizers and pesticides, are also increasing significantly, given that these are mainly imported from Russia. The knock-on effects will likewise worsen the food security situation in the near future. Thus, the topic of this special issue is even more important than it was when it was launched, especially as Tanzania is likely to be among the 10 largest countries globally in 2100 (8) and, simultaneously, climate change will hit East Africa (9) including Tanzania (10) particularly hard. The included set of papers aim to address the overall challenge of food insecurity from different disciplinary angles and on different analytical levels, thus holistically displaying the magnitude of the problem and showcasing potential pathways for solving some aspects of the crisis.

An often-overlooked aspect of food security (11), the adequate supply of cooking energy is addressed by Scheid et al., who apply an analytical framework to analyze the personally experienced impact of coping strategies when affected by reduced fuelwood supply. Thus, the paper provides an approach to develop a reliable monitoring framework of how adaptation measures are applied in response to fuelwood scarcity.

Schlindwein et al., in contrast, address, with a specific gender focused framework, another aspect of food security that is often neglected in respective discussions. The basic notions of masculinities and femininities of a specific inter-ethnic group, the Wagogo, are analyzed in the context of discourses on nourishment projects. In sum, the paper reinforces the need to address the ethnic identities of the researched regions in order to not just comprehend the traditions and culture, but also the food and nutrition of the indigenous people.

On a meso-scale, Bongole et al. develop a multinomial endogenous treatment effect model,focusing on the Tanzanian regions of Mbeya and Songwe, to evaluate the impacts of various combinations of climate-smart agricultural (CSA) practices on household food security. One major finding is that the highest payoffs regarding food security are achieved if CSA practices are used in combination, which is an important message for not just regional and national policymakers, but also the international donor community.

Habtemariam et al. focus on the link between improved household dietary diversity and diversified farm production. The analyses are based on four regression models with two different production diversity measures and two panel estimation methods. In three out of the four models, production diversity is significantly and positively correlated with a dietary diversity measure. Furthermore, the total crop and livestock species count show a significant positive association with dietary diversity. As a major finding, the authors underline the importance of a careful selection of appropriate production diversity indicators, which are in turn tailored to the specific circumstances of the local agricultural system.

A related study is by Kissoly et al., who scale out even further, comparing data from Tanzania and Kenya to analyze whether farm production diversity affects household diets. Their findings show that, in both countries, production diversity positively and significantly influences indicators of household dietary diversity, even though a more direct effect could be show in more rural areas. Furthermore, the paper shows that these effects decrease with increased market access. The authors underline the need for policymakers to consider context specific interventions when focusing on farm production diversity.

The papers in this special issue present a transdisciplinary and integrative view of food security and malnutrition in Tanzania. Food insecurity in Tanzania shows a very complex scenario. This complexity requires a deep knowledge of the local and regional environmental, socio-economic, and cultural systems. Discovering adequate technologies for more efficient agriculture production and energy conservation is crucial, but these are not alone enough. A vital element is the integration of existing local knowledge on innovative strategies and the active policymaker's involvement to ensure the development of policies that address the four pillars of food security.

## Author contributions

HH drafted the initial manuscript. All other authors contributed equally to finalization. All authors contributed to the article and approved the submitted version.

## Funding

This research was supported by funds of the Federal Ministry of Food and Agriculture (BMEL) based on a decision of the Parliament of the Federal Republic of Germany via the Federal Office for Agriculture and Food.

## Conflict of interest

The authors declare that the research was conducted in the absence of any commercial or financial relationships that could be construed as a potential conflict of interest.

## Publisher's note

All claims expressed in this article are solely those of the authors and do not necessarily represent those of their affiliated organizations, or those of the publisher, the editors and the reviewers. Any product that may be evaluated in this article, or claim that may be made by its manufacturer, is not guaranteed or endorsed by the publisher.
